# The Place of Medicine in Civilisation

**Published:** 1894-04-28

**Authors:** 


### The Hospital

An Institutional Journal of

The Medical Sciences and Hospital Administration, with

Vol. XVI.—No. 396.] AN EXTRA NURSING SUPPLEMENT. [April 28, 1894.

### The Place of Medicine in Civilisation.

The *Spectator* complains of the medical profession that though it knows all about foods and what the people might most profitably eat, it does not teach with enthusiasm, and the public is hardly any wiser in this fundamental and every-day question than its grandfathers were. It had been our opinion for a long time that the boot was on the other leg. Medicine has been accumulating all kinds of generally useful knowledge with extraordinary rapidity for the last half-century. That knowledge, being knowledge of man, material knowledge, knowledge of muscle and brain, of stomach and breathing apparatus, of liver and heart—knowledge of exactly those parts of man which most conserve his material interests and promote his mental contentment—one would have thought the modern public would have rushed at it with eagerness, and would have paid almost any price for it. But the facts are not so, however the *Spectator* may protest. The public takes our knowledge exactly as it takes our mixtures and pills, with a wry face, and as if it were something, though compulsory and inevitable, yet as nauseous as nauseous can be. Of this we feel that it is our right to complain. Notwithstanding all our efforts —efforts of the most scientific, honest, and unsurpassably important character—we are still treated as mere physiological watchdogs, body scavengers, occasional stomach helps, or liver comforters; but never, or hardly ever, as the guides, philosophers and friends of a materialising but only half comprehending generation.

Hundreds of proofs of this contention of ours could be adduced did space permit. Let us take one. It is an example of a doctor's bible, or at least of one sacred book belonging to a canon which is not yet closed, and which, we may devoutly hope, never will be closed. It is the Thirty-ninth Annual Report of the Registrar-General on the births, deaths, and marriages registered in Scotland. This book, which may be looked upon as an annual sermon, or if we like to go further back in history for a simile, as an annual prophesying, shows well, not indeed what the place of the doctor in modern civilisation is, but what it ought to be. The modern doctor is the prophet, priest, and teacher of the body. We are not forgetting that many intelligent individuals, just as they are their own prophets, priests, and guides in things spiritual, so are they in the things of the body. They need no physician, no lantern, no guide. To them the axiom "a man is either a fool or a physician at forty" is an axiom which they have proved in their own experience. But such individuals do not constitute the world: on the contrary, they are but as one in a million, or even in ten millions. The world of the present day, if it is to live according to knowledge, and according to the true, though late revealed, evangel of the body, not only needs, but should believe in, and honour, and seek the guidance of the prophets, priests, and teachers of the scientific lore of the body.

Not very many decades ago there were a class of religious propagandists who found that by far the most successful arguments they could use for the reformation of obstinately irreligious persons were what were called the "terrors of the lost." This annual report of the Scottish Registrar-General, when read with a seeing eye, is full of matter of such a minatory and terrifying kind. It has, moreover, this advantage over the speculative Gehenna of the enthusiastic theologian, that it deals with facts as solid, as stern, as real, and as demonstrable as are the rocks, or the sea, or the midday sun. Here, for example, is a fact of a very significant order to him who can understand. In the large towns of Scotland there are 2·16 persons in every hundred who die annually, whereas in the outlying country districts there are only 1·51 in every hundred. That is more than 21 in every thousand of the people of Glasgow and Dundee die every year, whereas only a little over 15 in every thousand die every year in the country districts. But the fact of death is only the indicating climax of hundreds of tragical stories of illness, pain, money loss, heartbreak, which have too often preceded these thousands of annual premature deaths. Now, here is a voice of terror, of thunder, of certain death, which warns mankind against leaving the open, pure air and healthy occupations of the country for the crowded and poisonous dwellings, dingy streets, and deadly industries of large towns. But who heeds the voice? It cannot be said that doctors have hidden this kind of light under a bushel. On the contrary, they have proclaimed it from practically every housetop in the civilised world. But with what result? With the result that hardly anybody, certainly not the daily journalist, not even the more serious weekly newspaper writer, ever thinks of reading, much less of making public, a single idea on the subject. The public desires lively copy: the newspaper writer finds the living, breathing, suffering, and dying realities of the Blue book deadly dull; and so he speaks no word of them—not one. This has been going on for several decades. Who can wonder then if the doctor, finding that his facts are treated not as stern scientific revelations, but as mere professional counters, ceases, in the eloquent language of an ancient reformer of men, to "cry aloud and lift up his voice like a trumpet' ?

One more fact of precisely like import may be used in illustration of the position here taken up. It relates to births, not to deaths. In the eight principal towns of Scotland the proportion of births to population is 3·25 to the hundred; in the remoter districts of the country it is only 2·18. Thatis for every three children, in proportion to population born in towns, there are not quite two born in the country. But what a significant fact this is to the political philosopher at the present moment! By mere numbers, in proportion to available area, our country is threatening to be swamped. But what is far more portentous than the mere increase of numbers is the fact that the town-born child is feebler in physique, feebler in brain, more excitable, less disciplined, much less likely to be religious; in short, a wilder, more uncontrollable, more exacting, and more dangerous political animal altogether than his country-bred brother. The difference between the country man and the town man is often this, that the former can give much of labour and defence, whilst he demands little; the latter can give little of labour and defence, but demands much. It is obviously, then, to the interest of a State like our own to promote the increase of population in the country, and to check the inordinate and dangerous rapidity of its increase in towns. All this the science of medicine, we ourselves among others, have pointed out again and again. It is far more important than the mere question of food, because men, although they may err in matters of diet, do not err deeply or dangerously. But this want of balance between the solid, muscular, slow, and trustworthy country element, and the excitable, frothy, and uncontrolable element of town, is, we consider, one of the most urgent and serious dangers of the time. Yet what newspaper preaches the gospel of making the country attractive, and so of building up a population like that of rural France, staid, stubborn, strong in muscle, and conservative to the backbone in the best of all senses? This is a mere hint on a subject on which volumes might be profitably written.

The medical knowledge of the present day is looked askance at by the public; and that because it is difficult and progressive. Scientific medicine is like religion, it makes high demands for itself; high demands upon the individual, of self denial, of rational conduct; high demands upon the State, of stern repression here and there, of resolute constructive activity. The medicine of to-day, the front rank medicine, is first and chiefly a scientific movement. It consists in the knowledge of man; the knowledge, that is to say, as distinguished from the results of introspection and speculation. Medicine for a hundred years has striven to know, and now she knows. Because she knows, she must lead, as all knowers in all ages have led; or if they have not, the loss has been not to themselves, but to those who have refused to be led. Medicine is, or strives to be, a real science of man. It is compelled to be this in modern times, in order that it may be competent in the healing art. Nothing less than a complete science of man enables us to be all round healers. But if our science be real science, and relatively complete as a science of man, must not all men follow our lead, or suffer the penalties that always and everywhere attach to error and to wilful ignorance?

